# Supplementary material for: A Spatial Analysis of the Spread of Hyperendemic Sporotrichosis in the State of Rio de Janeiro, Brazil
Source: J Fungi (Basel). 2022 Apr 23;8(5):434. doi: 10.3390/jof8050434 (PMC9145434; doi:10.3390/jof8050434)
Supplement: Supplementary file 1 [file jof-08-00434-s001.zip › jof-1682658-supplementary.pdf]

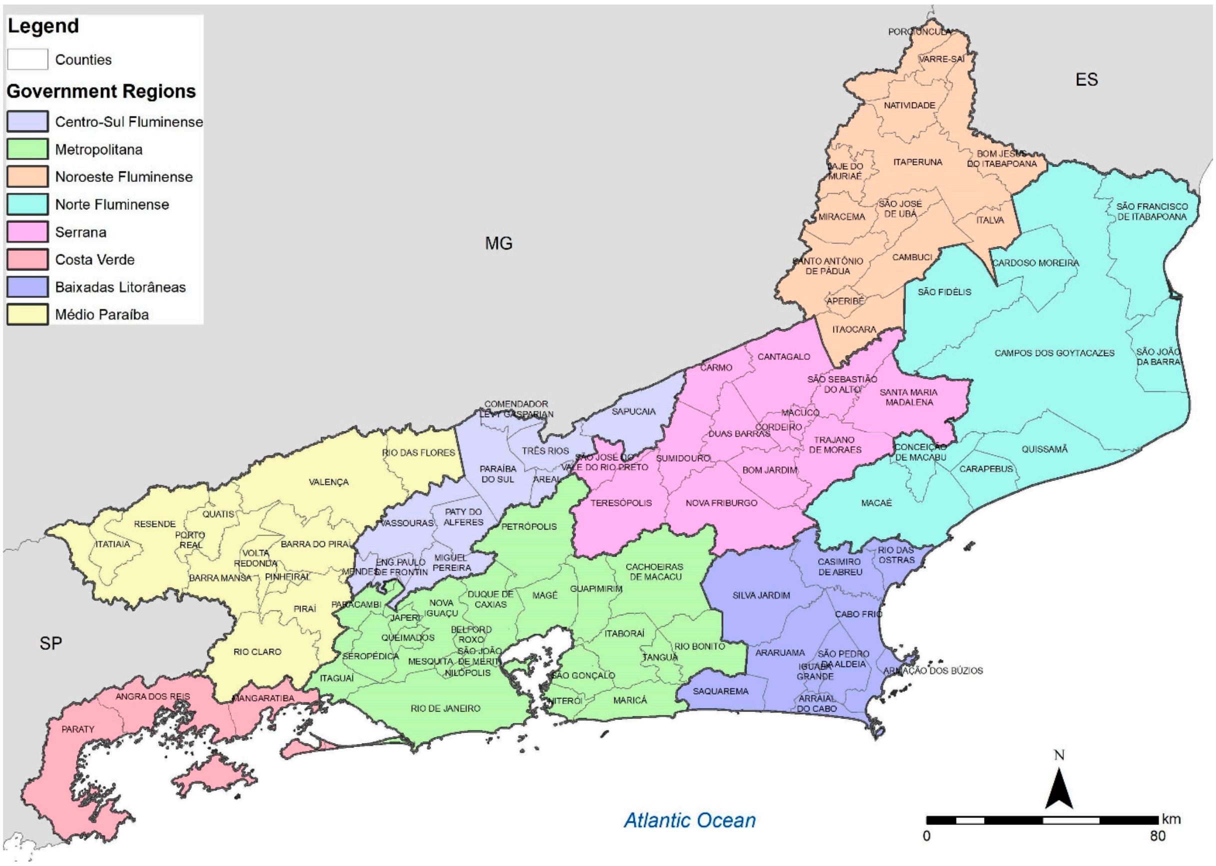

Supplementary Figure S1: Map of the state of Rio de Janeiro, its counties, and governmental regions. (Source: Limits of municipalities: Demographic Census 2010, IBGE. Governmental Regions: CEPERJ, 2018 (Fundação Centro Estadual de Estatísticas, Pesquisas e Formação de Servidores Públicos do Rio de Janeiro). Map created by the authors)

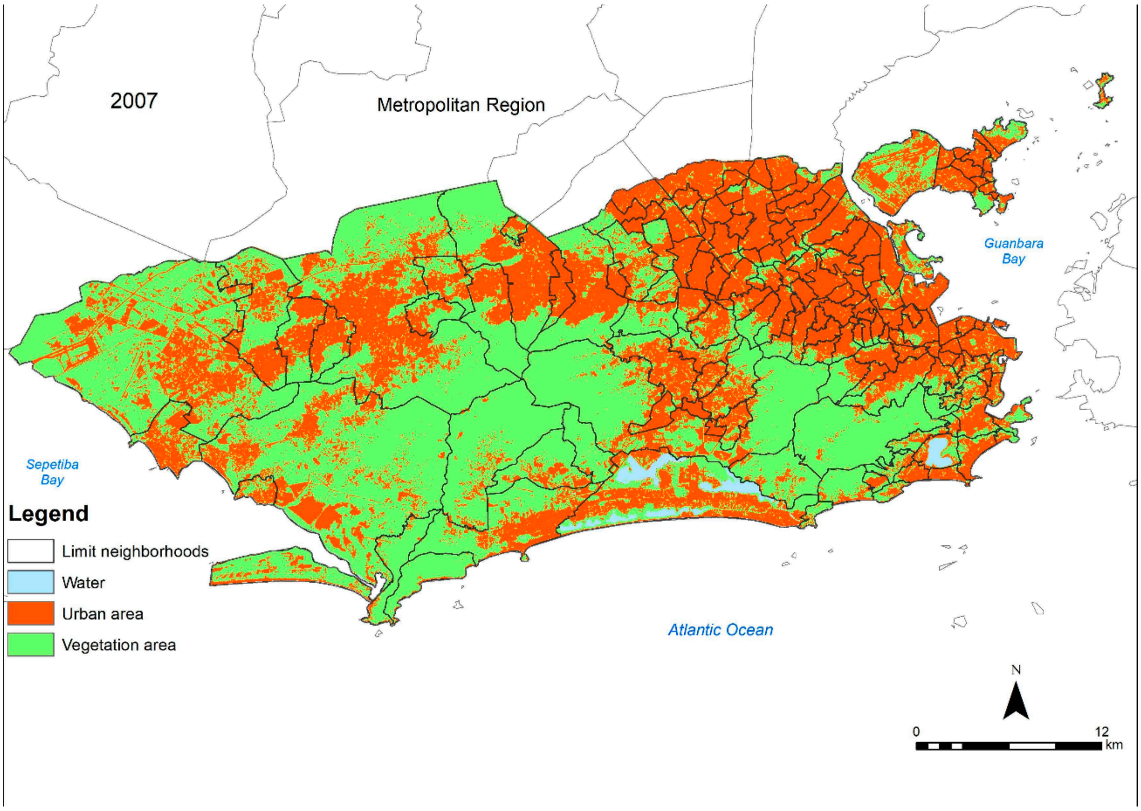

(**a**)

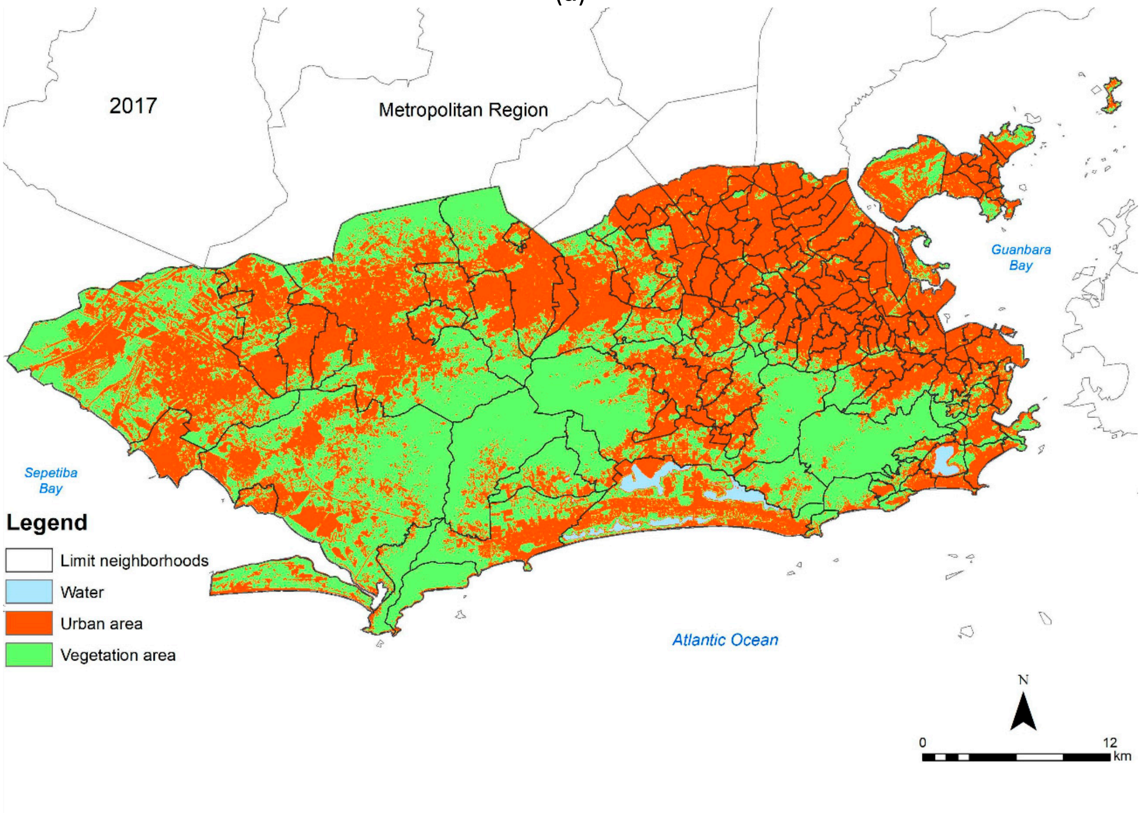

(**b**)

Supplementary Figure S2: Urban sprawl in the city of Rio de Janeiro, comparing the years (**a**) 2007 and (**b**) 2017 (Source: Satellite images (Landsat 5) provided by the National Institute for Space Research. Limits of neighborhoods: Demographic Census 2010, IBGE. Map created by the authors)

Supplementary Table S1: Socioeconomic and demographic characteristics of the regions of the state of Rio de Janeiro, Brazil.

Region of the state	Socioeconomic and demographic characteristics
Metropolitana (Metropolitan region)	Most of the state's industries are located there, forming a very diversified industrial park. It also brings together highly specialized services in the financial, commercial, educational and health sectors, as well as public bodies and institutions, among others. More than 70% of the population of the State is located there.
Baixadas Litorâneas (Coastal Shore region)	Until the 1960s, the region's economy was related to salt exploration, orange production, fishing and cattle raising. In recent decades, activities related to tourism and leisure have become more important in the region, with an increase in urbanization and the proliferation of housing in areas of environmental protection, among others.
Noroeste (Northwest Fluminense region)	The region´s economy is historically based on the production of coffee and agriculture. The misuse of land and extensive cattle raising has been causing a decrease in the number of rural populations.
Norte Fluminense (Northern Fluminense region)	The region's economy is based on the sugar agribusiness. In recent decades, oil and natural gas have assumed a significant role in the regional economy and have led to an accelerated and disorderly urban growth.
Serrana (Mountain region)	The region is composed of two different spatial portions. One characterized by intense industrial and tourist activity, in addition to the production of vegetables and textiles. The other has a weak economic performance and low productivity rates, which has served to force a reduction in the rural workforce.
Centro-Sul Fluminense (Central South Fluminense region)	The region´s economy is based on cattle raising, horticulture and tourism.
Médio Paraíba (Middle Paraíba region)	After the Metropolitan region, it is the most industrialized region in the state of Rio de Janeiro. Industrialization generates the expansion of sub-housing and under-equipped peripheries. Agriculture is also important, and the region is one of the largest milk producers in the state.
Costa Verde (Green Coast region)	Tourism is the main activity in the region, due to its natural beauty. In addition, agricultural activities such as the cultivation of bananas and fishing are important in the region. Local ecosystems have been degraded by real estate activity.

Modified from CEPERJ – Coordenadoria de Geociências (Available online: https://www.ceperj.rj.gov.br/?page_id=262)
